# Fasting reduces satiety and increases hunger but does not affect the performance in resistance training

**DOI:** 10.5114/biolsport.2024.131814

**Published:** 2023-04-10

**Authors:** Marcos D.M. Drummond, Paula S.G. Soares, Lucas A. Savoi, Ronaldo A.D. Silva

**Affiliations:** 1 Laboratory of Nutrition and Sport Training, Federal University of Minas Gerais, Belo Horizonte, Brazil

**Keywords:** Fasting, Resistance training, Athletic performance, Hunger

## Abstract

Intermittent fasting (IF) has been suggested to reduce body fat percentage and improve non-communicable chronic diseases. However, little is known about resistance training (RT) and the subjective perception of hunger under fasted conditions. This study aimed to examine the effects of overnight fasting (12 h or 16 h fasting) on the maximum voluntary isometric contraction (MVIC) and countermovement jump (CMJ) performance in resistance-trained young male adults. In RT sessions, the maximum number of repetitions (MNR) and the total volume load (TVL) were evaluated in the back squat and leg press 45°. The volunteers performed all tests and the RT session in 3 different conditions: fed state, 12 and 16 hours of IF. The subjective perception of hunger was applied through an adapted visual analogue scale (adVAS). The results showed that strength and power variables did not change significantly: MVIC (p = 0.960), CMJ (p = 0.986), MNR back squat (p = 0.856), MNR leg press 45° (p = 0.998), TVL (p = 0.954). However, hunger was significantly greater after the 16-hour fasting (p = 0.001) compared to 12 hours of fasting and the fed state. Also, the desire to eat was greater after 16 hours (p = 0.001) compared to 12 hours of fasting and the fed state. This study indicates that IF for 12 or 16 hours does not significantly impair strength and power, but the longer the fasting duration, the greater are the hunger and desire to eat.

## List of abbreviations

adVAS: Adapted visual analogue scale
CMJ: Countermovement jump
FS: Feed state
IF: Intermittent fasting
IF-12: Fasting 12 hours
IF-16: Fasting 16 hours
MNR: Maximum number of repetitions
MVIC: Maximum voluntary isometric contraction
ND: Normal diet
RPE: Rating of perceived effort
s-RPE: Session rating of perceived effort
RT: Resistance training
TVL: Total volume load
PAR-Q: Physical Activity Readiness Questionnaire
1RM: One repetition maximum test

## INTRODUCTION

Intermittent fasting (IF) is a recurrent eating pattern with energy restriction interspersed with periods of *ad libitum* food intake [1]. Some authors have suggested that IF induces metabolic adaptations characterised by the mobilisation and utilisation of stored fat as an energy source (lipolysis), which have been associated with favourable effects on insulin sensitivity, reducing fat mass and improving some markers of non-communicable chronic diseases [2]. The 16/8 IF protocol (16 consecutive hours of fasting and 8 hours of eating) may be the most effective and sustainable because it is the caloric restriction most closely to the human dietary pattern [2, 3]. The 16/8 IF became popular as the basis for the “Leangains Diet”, which associates this nutritional strategy with resistance training (RT) [4]. RT is performed to preserve and mitigate losses of muscle mass caused by caloric restriction [5, 2], impairment of muscle strength and reduced training performance proportionally to the duration of fasting [5]. Such deleterious effects can occur due to the reduction in muscle glycogen stores and glycaemia, with an increase in the rating of perceived effort (RPE) [6, 2]. However, the few studies that associated resistance training and 16/8 IF [3, 7, 8] claim that body composition, especially lean mass and strength performance, is not negatively affected by IF 16/8. However, these studies performed the strength test sessions and the RT in the fed state. In contrast, Naharudin et al. [9] discovered that fasting reduces the maximum number of repetitions (MNR) acutely performed in the back squat during the fasting state (10–13 h overnight) in trained individuals who skipped breakfast. Some studies identified bad mood and reduction in general disposition, motivation and exercised enjoyment in training in fasting conditions [7, 10, 11]. Diminished levels of blood glucose and increased cortisol levels may be considered the main factors contributing to the negative affective state and the decrease in overall mood and pleasure experienced during physical exercise while fasting [7, 10, 11]. Such factors could limit adherence to IF in the long term. However, in an investigation lasting 4 weeks, Stratton et al. [7] did not identify a significant impact of IF on subjective perceptions of fullness, desire to eat, or hunger after 8 hours of IF before the RT session. Furthermore, no other studies were found that investigated subjective perceptions of hunger and fullness during fasting in RT.

Therefore, the acute effect of fasting on strength performance, that is, on the ability to perform training sessions during the fasting period, has not yet been evaluated, even though a reduction in physical performance is expected to be proportional to the length of fasting time [5]. However, no studies have compared performance, hunger and fullness perceptions in RT at different fasting times, which can also interfere with diet and training plan adherence.

Thus, the objective of the present study was to verify the performance of trained individuals and the subjective perceptions of hunger and satiety, in the fasting state, after 12 and 16 hours. The hypotheses were that acute fasting would negatively influence performance and increase subjective perceptions of hunger and satiety proportionally to the fasting time.

## MATERIALS AND METHODS

### Participants

The volunteers were 16 men (24.9 ± 3.12 years; 79.2 ± 6.42 kg; 1.76 ± 0.05 m; fat mass percentage of 18.4 ± 4.87%). The total RT experience of the volunteers was 2.9 ± 0.6 years, and the mean relative maximum lifted mass (1RM) in the squat was 2.14 ± 0.47 kg. Furthermore, the volunteers were experienced in performing the back squat and leg press 45° exercises with good technique and concentric failure. Therefore, they are classified as trained [12].

This study included only eutrophic individuals who were not undergoing any nutritional intervention and had not consumed any nutritional ergogenic resources for at least 30 days prior to the study. Additionally, participants were required to have reported no joint or muscle injury within six months of the study and to have answered “no” to all questions on the Physical Activity Readiness Questionnaire (PAR-Q), a questionnaire that assesses the safety and risks of physical activity.

The sample size was determined based on the effect size of fasting on strength performance, as reported by Naharudin et al. [9]. Consequently, we recruited the exact number of participants required for the calculation of the sample size, considering an effect size of 0.98, power of 0.95, and alpha level of 0.05. The search for volunteers was carried out by publicising the study and its inclusion criteria in the institution’s regional training centres, social networks and research team. Upon reaching the expected number, the volunteer’s search was interrupted. There was no exclusion of participants before, during or after the study. All volunteers signed the Participant Consent Form agreeing to participate in the study. This study was submitted to the Ethics and Research Committee of the Federal University of Minas Gerais (CAAE: 47069421.0.0000.5149). The approval protocol number is 4.389.077.

### Procedures

In this study, the acute assessment of single and non-consecutive days of IF 16/8 and IF 12/12, classified as time-restricted feeding, was performed. Thus, the IF protocols consisted of a window of 8 or 12 hours of ad libitum feeding interspersed with a period of 16 or 12 hours without ingesting food, caffeine or stimulants, respectively [1, 7].

The volunteers were trained to register all foods consumed and their portions in the FT app through descriptions and photographs. On the day of the experimental testing and training sessions, the reports were reviewed to ensure accordance with the fasting protocol. All sessions were performed in the morning. The schedules and the last meal respected the volunteers’ preferences.

The fasting experimental conditions were:

– Fed state (FS): strength tests and RT session in the morning, one hour after breakfast ingestion, as identified in the FT of experimental session three.
– Fasting 16 hours (IF-16): breakfast omission on the session day and starting the test session after 15 hours of fasting, concluding 16 hours of fasting at the end of the session.
– Fasting 12 hours (IF-12): breakfast omission on the day of the session and starting the test session after 11 hours of fasting, concluding 12 hours of fasting at the end of the session.

### Experimental design

This study was a randomised crossover clinical trial. The IF was characterised by 12 and 16 hours of overnight fasting. Initially, in the first experimental session, the study was presented to all participants along with a demonstration on how to use the app for food tracking (FT). At the demonstration, the researchers guided the participants to download the food tracking app onto their smartphones and enter their personal information, such as height, weight and age. They also explained how to log all foods and beverages consumed throughout the day, including portion sizes, at the appropriate times. Also, the body composition was determined along with the Physical Activity Readiness Questionnaire (PAR-Q). Total body mass and composition were assessed using an Inbody 270 bioimpedance scale (Inbody Co., Ltd., Korea) and height using a portable stadiometer. Participants were advised to wear lightweight clothing, remove metal objects, stay well hydrated for 24 hours, urinate at least 30 minutes before the assessment, and abstain from alcohol and caffeine. Additionally, all participants were assessed in the FS but were advised to avoid eating a large meal and engaging in strenuous exercise in the 24 hours preceding the assessment. Furthermore, the volunteers were instructed to maintain their usual eating habits throughout the study period, which was verified through food recall and photographic records. Therefore, participants had either the same dinner the night before the intermittent fasting protocol or their regular breakfast as usual.

In the second experimental session, the volunteers performed a familiarisation session of the equipment and procedures used in the tests and RT session. Then, in experimental session three, they performed the one-repetition maximum test (1RM) in the back squat and in experimental session four, they performed the same test in the leg press 45°. This test was performed to prescribe the intensity of the RT session [12].

The fifth to the seventh experimental sessions were designed for strength and power tests, maximum voluntary isometric contraction (MVIC) and the countermovement jump (CMJ), respectively, in addition to the RT in one of the three experimental conditions: fed state (FS), 12 hours of fasting (IF-12) and 16 hours of fasting (IF-16).

The subjective sensations of hunger, satiety and glycaemia were collected before and after the strength and power tests and after RT. In addition, the lactate concentration was registered before the tests and after the experimental session. The RPE was collected 60 seconds after each series of exercises, and the session-RPE was collected 30 minutes after the end of the experimental sessions. The volunteers used the FT app Dietbox smartphone (Dietbox, version 7.9.0., Brazilian food composition table) one day before experimental sessions three to seven.

**FIG. 1:**
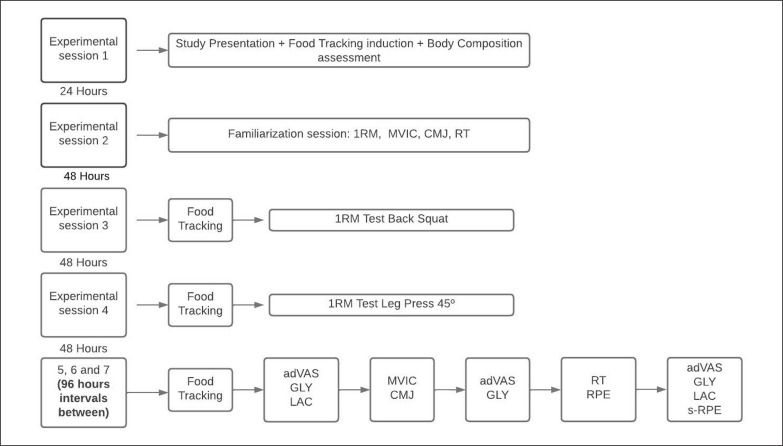
Study design. 1RM: 1 Repetition Maximum Test; MVIC: Maximum Voluntary Isometric Contraction Test; CMJ: Countermovement Jump Test; FT: Food Tracking; adVAS: adapted Visual Analogue Scale; RT: Resistance Training; GLY: Glycemia; LAC: Lactate concentration; RPE: Rating Perceived Effort; s-RPE: Rating Perceived Effort ater the end of the session.

Between experimental sessions one and two, there was a 24-hour pause. Therefore, the intervals between experimental sessions two, three, four and five were at least 48 hours. Between experimental sessions five, six and seven, intervals of at least 96 hours were used to prevent a possible effect of fasting from accumulating and promote complete recovery between experimental conditions [13]. The study design is presented in Figure 1.

### Performance in experimental sessions

The physical performance assessment consisted of the MVIC tests for the back squat [14] and the CMJ test [15]. The total load volume (TVL) (total number of repetitions × weight lifted) was used to monitor the performance of RT sessions [16]. The interval between tests was 5 minutes and 10 minutes between the last test and the RT session. In the RT session, five sets were performed with repetitions until concentric failure, at 70% of 1RM in the back squat and leg press 45° exercises. The pause between sets and exercises was 2 minutes. In addition, the first series of each exercise was used as a reference for evaluating and comparing MNR performed between experimental conditions [7].

### Strength and power tests

The back squat and leg press 45° techniques in the tests and RT were standardised, similar to the study by Claudino et al. [15]. Adjustments were allowed according to the volunteers’ preferences and experiences, recorded and repeated in all experimental tests and training conditions. The experimental sessions to familiarise the participant’s strength tests (1RM and MVIC) was the same protocol followed by Drummond et al. [14]. The power was assessed through the CMJ test and its familiarisation [15].

The 1RM test protocol was performed following what was proposed by Drummond et al. [14]. The MVIC test consisted of 3 maximal voluntary isometric actions in back squat lasting 6 seconds with a 3-minute interval between actions. The position was standardised with knee and hip flexion at 90º, determined by the Angle Meter app (version 1.9, iOS). The highest strength value was recorded for comparison [14]. This test was performed on a force platform, model PLA3–1D-7KN/JBA (ZbStaniak; Warsaw, Poland, 1 N precision), connected to a computer interface with the MAX5 program (version 5.1; JBA, ZbStaniak), which presented the force curve as a function of the time [14].

The CMJ protocol followed the procedures suggested by Claudino et al. [15]. Participants completed a 5-minute warm-up consisting of cycling and dynamic stretching. Subsequently, they stood still with their hands on their hips and flexed their knees to approximately 90 degrees. After a brief pause, they executed a maximal vertical jump, aiming to maintain their legs extended and their arms static throughout the jump. The volunteers performed a single set of four consecutive jumps. The CMJ was performed on the same MVIC force platform. The means of peak power in watts (W) and maximum height in centimetres (cm) of the jumps were recorded and compared in each experimental condition [12].

### Subjective perceptions of hunger and satiety

The present study assessed subjective perceptions of hunger and satiety using an adapted visual analogue scale (adVAS) [17]. Aiming to facilitate the visual analysis of the volunteers, registering and analysing the answers, a scale from 1 to 10 was adapted and standardised following a visual scale with answers anchored in the initial and terminal portions that describe the extreme sensations. For instance: 1 = “no, not at all”; 10 = “yes, a lot”). The participants were instructed to verbally communicate their perceptions so that the quantitative answer could be recorded. Before, during, and after the experimental session, the adVAS variables “hunger,” “desire to eat,” and “fullness” were recorded.

The subjective perceptions of hunger and satiety were used as an assessment parameter of hunger itself, while the desire to eat and fullness were used as subjective parameters of satiety [7, 17].

### Rating of perceived exertion (RPE)

The RPE was collected 60 seconds after the end of each series of exercises. The familiarisation with RPE was performed in the experimental sessions of the 1RM test. In addition, 30 minutes after the end of the training session, the volunteers were asked about their perception of the training session exertion (session-RPE) without the acute session fatigue. On the scale proposed by Foster et al. [18], the volunteers indicated the value corresponding to the perceived exertion in the test session and training. Session-RPE was calculated from the Borg CR-10 scale × training duration (minutes) [18, 19].

### Glycaemia and lactate concentration

The glycaemia measurement was used in this study to ensure that the volunteers were not in a hypoglycaemic state before starting the test and training sessions [19]. Moreover, glycaemia and lactate concentration response were investigated to analyse the use of energy substrates during effort [12, 20].

Glycaemia was also determined at the end of the tests and after the resistance training session to monitor this parameter’s behaviour between the participants. The glucometer used was the FreeStyle model (Abbott, Brazil). In addition, blood lactate was measured before and after the experimental session (after 3 minutes of rest) [21] with the Accutrend lactometer (Roche Diagnostics, Switzerland). The procedures adopted were the same as those used by Franchini et al. [20].

### Statistical analysis

The data were expressed as mean ± standard deviation. Data normality was analysed using the Shapiro-Wilk test, and homogeneity of variances using the Levene test. The ANOVA one-way repeated measures test was used to compare the variance of the physical performance of individuals in the strength, power test sessions, resistance training, RPE, session-RPE and the appetite parameters investigated through the adVAS in the conditions FS, IF-12 and IF-16. When the variables did not present a normal distribution, Friedman’s non-parametric equivalent test was used. The ANOVA two-way test for repeated measures was used to analyse the glycaemia and lactate concentration variance. Friedman’s non-parametric equivalent test was used to analyse the times when the adVAS was applied during the experimental sessions. A significance level of 0.05 was adopted, and the Bonferroni post hoc test was used when necessary. The epsilon (ε²) and eta squared (η²) effect sizes were calculated and classified as trivial(< 0.004), small (0.004–0.25), moderate (0.025–0.64) or strong (> 0.64) [22]. Data analysis was performed using the statistical software Jamovi 1.6.23.0.

### RESULTS

**FIG. 2:**
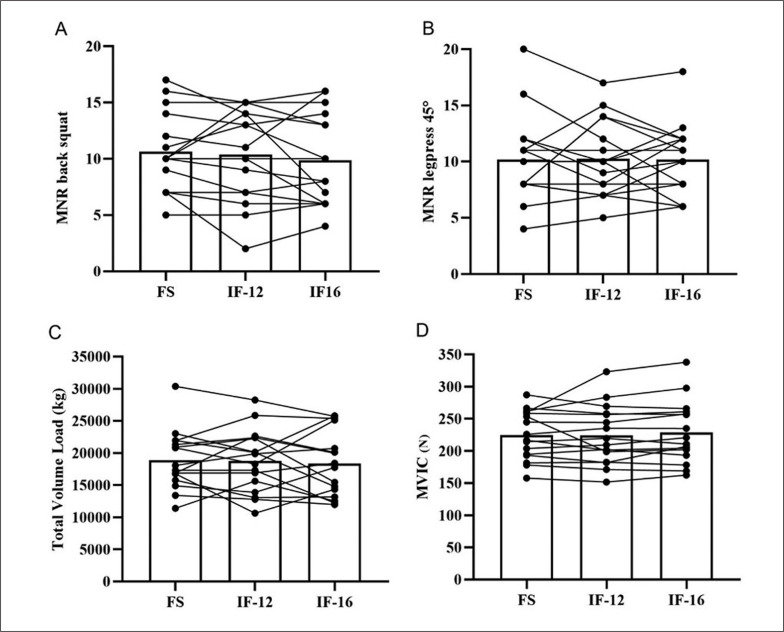
Individual MNR variations in the back squat (A), leg press 45° (B), training TVL (C) and MVIC test (D) in relation to three different feeding conditions.

The MVIC, training session performance, glycaemia and lactate concentration results showed normal distribution and verified homogeneity. In contrast, session-RPE, back squat and leg press 45° RPE did not show normal distribution. In the CMJ, no statistically significant differences were identified in the mean maximum height (cm) and peak power (W) between the experimental conditions (p > 0.05) (Table 1). The strength performance (N) in the MVIC test was similar in the three experimental conditions (p = 0.960; F = 0.332) (Table 1). The MNR in both exercises and the TVL of the training sessions were also similar between the three experimental conditions (Table 1). The individual performances in the MVIC test, MNR and TVL are shown in Figure 2.

**TABLE 1:** Performance in tests and resistance training session (mean ± standard deviation)

	FS	IF-12	IF-16	p-value	F	η²	classification (η²)
**CMJ (cm)**	35.2 ± 0.04	35.2 ± 0.04	35.4 ± 0.04	0.986	0.015	0.001	Trivial
**CMJ (W)**	1880 ± 263.13	1945.03 ± 291.90	1914.63 ± 369.69	0.807	0.215	0.008	Trivial
**MVIC (N)**	224.84 ± 38.09	224.28 ± 45.86	228.65 ± 48.14	0.960	0.332	0.002	Trivial
**TVL**	18852.24 ± 4541.88	18749.35 ± 4897.08	18337.33 ± 5124.48	0.954	0.048	0.002	Trivial
**MNR Back Squat**	10.63 ± 3.44	10.38 ± 4.06	9.88 ± 4.06	0.856	0.156	0.007	Trivial
**MNR Leg press 45°**	10.19 ± 3.82	10.25 ± 3.38	10.19 ± 3.17	0.998	0.002	0.000	Trivial

CMJ: Countermovement Jump; cm: Centimetres; W: Watts; N: Newton; MVIC: Maximum Voluntary Isometric Contraction; TVL: Total Volume Load; MNR: Maximum Number Repetition; FS: Feed State; IF-12: 12-hour Intermittent Fasting; IF-16: 16-hour Intermittent Fasting; F: ANOVA One-way repeated measures; η²: Effect Size;

Hunger was significantly greater in the 12-hour fasting (pre: p < 0.001, t = -7.903; during: p < 0.001, t = -5.229; post-session: p < 0.002, t = -4.389) and 16-hour fasting (pre: p < 0.001, t = -8.950; during: p < 0.001, t = -6.157; post-session p < 0.001, t = -5.467) compared to the fed state. However, there was no statistically significant difference in hunger between the 12-hour and 16-hour fasting at any time (pre: p = 1.000, t = -1.047; during: p = 1.000, t = -0.928; post-session: p = 1.000, t = -1.078) (Table 2).

**TABLE 2:** Moments of collection of adVAS parameters concerning feeding conditions, Δ (% fasting12h/fed), Δ (%fasting16h/fed), effect size and classification (ε²)

adVAS	FS	IF-12	Δ (%)	IF-16	Δ (%)	ε²	Classification (ε²)
(Hunger – Pre)	1.44 ± 0.73	6.63 ± 2.03^*^	460.42	7.31 ± 2.39^*^	507.64	0.68	Strong
(Hunger – During)	2.38 ± 1.50	6.25 ± 2.35^*^	262.61	6.94 ± 2.32^*^	291.60	0.52	Moderate
(Hunger – Post)	3.19 ± 2.29	6.75 ± 2.52^*^	211.60	7.63 ± 2.06^*^	239.18	0.41	Moderate
(Desire to eat – Pre)	3.50 ± 1.97	7.38 ± 1.86^*^	210.86	8.44 ± 1.46^*^	241.14	0.58	Moderate
(Desire to eat – During)	4.00 ± 2.28	7.31 ± 1.89^*^	182.75	8.19 ± 1.68^*^	204.75	0.45	Moderate
(Desire to eat – Post)	4.00 ± 2.13	6.69 ± 2.52^*^	167.25	7.81 ± 1.97^*^	195.25	0.35	Moderate
(Fullness – Pre)	7.31 ± 1.82	2.81 ± 1.80^*^	38.44	2.38 ± 1.50^*^	32.56	0.59	Moderate
(Fullness – During)	7.31 ± 1.45	3.06 ± 1.81^*^	41.86	2.25 ± 1.18^*^	30.78	0.64	Strong
(Fullness – Post)	6.56 ± 2.16	3.19 ± 2.10^*^	48.63	2.63 ± 1.93^*^	40.09	0.41	Moderate

* Statistically significant differences of parameters adVAS in the fed condition compared to 12-hour fasting (IF-12) and 16-hour fasting (IF-16) conditions; FS: Feed State; Δ: Difference; ε²: Effect Size

The desire to eat was significantly greater in the 12-hour fasting (pre: p < 0.001, t = -6.177; during: p < 0.001, t = -4.766; post-session: p < 0.048, t = -3.423) and in the 16-hour fasting (pre: p < 0.001, t = -7.870; during: p < 0.001, t = -6.025; post-session: p < 0.001, t = -4.586) compared to the fed state. However, there was no significant difference in the desire to eat between the 12-hour and the 16-hour fasting (pre: p = 1.000, t = -1.694; during: p = 1.000, t = -1.256; post-session: p = 1.000, t = -1.433). Finally, it was identified that the fullness in the moments before (p < 0.001, t = 7.442), during (p < 0.001, t = 8.009) and after a session (p < 0.001, t = 4.260) was greater in the fed condition compared to the 12-hour fasting and the 16-hour fasting (pre: p < 0.001, t = 8.166; during: p < 0.001, t = 9.540; post-session: p < 0.048, t = 5.390). No significant differences were found in fullness between the 12-hour and the 16-hour fasting (pre: p = 1.000, t = 0.724; during: p = 1.000, t = 1.531; post-session: p = 1.000, t = 0.770) (Table 2).

In the back squat, the mean RPE was similar (p = 0.951, χ^2^ = 0.101) between the conditions (FS:8.41 ± 1.08; IF-12: 8.34 ± 1.04; IF-16: 8.55 ± 1.10). The effect size was trivial (ε² = 0.00261). Likewise, the RPE in leg press 45° was also similar (p = 0.654, χ^2^ = 0.848) in the experimental conditions (FS: 8.84 ± 0.936; IF-12: 8.86 ± 0.726; IF-16: 9.09 ± 0.906), with a trivial effect size = (ε² = 0.01804). The session-RPE values were also similar in the experimental condition (FS: 250 ± 34.9; IF-12: 252 ± 27.4; IF-16: 256 ± 28.5), without statistically significant differences (p = 0.913, χ^2^ = 0.182) and also with a trivial effect size (ε² = 0.00387).

The glycaemia was similar before, during and after the experimental session (p = 0.236; F = 1.47) in all conditions, with trivial effect sizes (η² = 0.012). As for the interaction between the times of glycaemia measurement and for each different condition, no statistically significant difference was found (p = 0.056; F = 2.40), and the effect size was considered trivial (η² = 0.038) based on the 95% confidence interval (CI) (Table 3). The initial lactate concentration was similar in the three evaluated experimental conditions (p = 0.2; F = 1.67). However, the lactate concentration was higher in the post-session compared to the pre-session (p < 0.001) in all experimental conditions, with a trivial effect size (η² = 0.01), considering a 95% confidence interval (CI) (Table 3).

**TABLE 3:** Glycemia and lactate concentration results.

	FS	CI 95%	IF-12	CI 95%	IF-16	CI 95%
Glycemia (Pre)	98.1 ± 9.75	93.4–102.9	89.9 ± 9.62	85.1–94.6	88.9 ± 9.05	84.1–93.6
Glycemia (During)	95.6 ± 8.87	91.3–99.8	86.2 ± 7.48	82.0–90.4	90.1 ± 8.76	85.9–94.4
Glycemia (Post)	90.1 ± 11.8	84.9–95.2	88.4 ± 7.10	83.2–93.5	90.8 ± 11.2	85.6–96.0
Lactate (Pre)	1.94 ± 0.98	1.45–2.44	2.17 ± 1.24	1.68–2.67	1.56 ± 0.613	1.06–2.05
Lactate (Post)	11.3 ± 2.60	9.43–13.2	12.2 ± 4.20	10.34–14.1	10.2 ± 4.06	8.36–12.1

FS: Feed State; CI: Confidence Interval; IF-12: 12-hour Intermittent Fasting; IF-16: 16-hour Intermittent Fasting.

## DISCUSSION

The present study results showed that the performance in the RT session and strength tests did not change significantly in any of the fasting conditions compared to the fed state. However, the perception of fullness significantly decreased, and hunger increased, similarly in the fasting conditions. Therefore, the hypotheses of the study were partially confirmed.

In studies with trained males submitted to eight and four weeks of intervention respectively, Moro et al. [23] and Stratton et al. [7] compared the results of maximal strength of an RT programme associated with IF 16/8 and a normal diet (ND). The performance improved at the end of the intervention, but no significant differences were found between the conditions. Moro et al. [24], who followed the same volunteers as Moro et al. [23] for 12 months, reached the same conclusion. These results indicate that IF 16/8 did not influence strength training, similarly to the present study. Tinsley et al. [25] evaluated and compared the performance of MVIC of women submitted to RT plus IF 16/8 and ND for eight weeks. A significant and similar increase was identified after the intervention in both conditions. In the present study, maximal MVIC strength was acutely assessed on single days in a 12-hour or 16-hour fast and showed no significant differences between the fasting conditions and the fed state. However, in the present study, the chronic effect of RT was not investigated. Only the RT session performance was evaluated and compared.

The present study also assessed jump height (cm) and peak power (W) of the countermovement jump (CMJ) and did not identify significant differences in either of these parameters between the different feeding conditions. These findings are consistent with previous studies conducted by Tinsley et al. [25, 26] and Stratton et al. [7]. The improvement in strength performance in RT associated with IF was principally due to neuromuscular adaptations to the RT protocol, and possibly the IF did not negatively interfere with this adaptation process [3, 7, 25, 26].

However, the studies that investigated RT associated with IF only performed and assessed the strength tests in the fed state when the volunteers did not perform fasting. Thus, to the author’s knowledge, the acute effect of IF on strength performance has not been investigated. This partially limits the discussion of the results. New studies are needed to investigate the acute and chronic effects on strength when strength tests and RT are performed during fasting.

The training performance parameters TVL and the MNR in back squat were also not influenced by the acute fasting of 12 or 16 hours in the present study. Contrary to these findings, Naharudin et al. [9] found an increase of approximately 15% in the MNR in a back squat in the fed state compared to overnight fasting (10 h–13 h fasting). However, in a subsequent study, where the same volunteers were submitted to the same fasting protocol, Naharudin et al. [10] found that MNR in back squats was higher in placebo conditions than control conditions, with no significant repetitions between carbohydrate and viscous placebo ingestions. Therefore, fasting would not have interfered with performance in the RT session, corroborating the present study’s findings.

In all experimental conditions, the maintenance of glycaemic levels was observed in the present study during and after the RT session. Thus, a potential daily oscillation range of liver glycogen during fasting and feeding periods had no deleterious impact on RT performance or glycaemic levels [10]. Furthermore, the RT session alone may not deplete substantial muscle glycogen when individuals recover between training sessions after a night’s sleep [10]. The present study results indicate that muscle glycogen stores were sufficient for RT session demands, which would explain the similar performance between the experimental conditions.

The lactate concentrations, characteristic of the proposed RT protocol, were similar in all conditions. Thus, fasting did not influence production and utilisation of this metabolite. Therefore, since the MNR and TVL did not undergo significant changes, the hypothesis that the primary energy source used in the RT session came from previous muscle glycogen stores is reinforced.

The results showed that the perception of hunger and the desire to eat were significantly higher in the fasting conditions. On the other hand, the feeling of fullness was lower in the fasting condition compared to the fed state. However, the altered fasting appetite perceptions were not sufficient to negatively impact strength and power performance.

The RPE and session-RPE remained high without significant differences in all experimental conditions, even with increased hunger and reduced satiety. These results corroborate the maintenance of performance in the evaluated conditions. No other studies have investigated the effect of IF on RPE in RT, whether acute or chronic, but it is still worth pointing out that the volunteers were verbally motivated to perform at maximum during the RT sessions and power and strength tests, which could justify the maintenance of RPE at high levels in all conditions, even if the participants were uncomfortable with the feeling of hunger. However, this might obfuscate the volunteer’s actual performance under routine training. Nevertheless, it is recommended that in subsequent studies, the volunteers receive no motivation during the training session in order to more accurately represent their reality during training and, consequently, their performance.

Slater et al. [11] found that fasting before evening exercise was associated with reduced motivation and exercise enjoyment. Furthermore, Naharudin et al. [10] and Stratton et al. [7] identified a reduction in general disposition and poor mood in fasting conditions. These factors can also influence adherence to diet and training planning, especially in RT. Thus, new investigations on the effects of IF on hunger, mood, fullness, and motivation should be conducted.

Among other limitations that have been previously mentioned, it is important to consider some factors that may reduce the applicability and reliability of the study when interpreting the results. One of these limitations is the use of the FT app to control and monitor food intake. Despite the tool’s limitations, the researcher took measures to ensure that the participants adhered correctly to the stipulated fasting protocol. This included sending individual and group reminders via mobile phone the day before data collection and reinforcing the guidelines on the day of the sessions. Controlling the repetition time was another possible improvement. This would be done to minimise external interference with the regular movement pattern of the volunteer.

## CONCLUSIONS

Our findings suggest that IF may not influence the performance of maximum strength, power, and the performance of an RT session, regardless of the fasting period. Consequently, resistance-training males, their coaches, nutritionists and other associated professionals can choose fasting for 12 or 16 hours as a nutritional strategy without negatively influencing strength performance.

However, the reduction in the subjective perceptions of satiety and the increase in hunger can make it challenging to adhere to training and diet planning, reducing their effectiveness.
